# Canagliflozin, an Inhibitor of the Na^+^-Coupled D-Glucose Cotransporter, SGLT2, Inhibits Astrocyte Swelling and Brain Swelling in Cerebral Ischemia

**DOI:** 10.3390/cells12182221

**Published:** 2023-09-06

**Authors:** Bosung Shim, Jesse A. Stokum, Mitchell Moyer, Natalya Tsymbalyuk, Orest Tsymbalyuk, Kaspar Keledjian, Svetlana Ivanova, Cigdem Tosun, Volodymyr Gerzanich, J. Marc Simard

**Affiliations:** 1 Department of Neurosurgery, University of Maryland School of Medicine, Baltimore, MD 21201, USA; bosung.shim@som.umaryland.edu (B.S.); jstokum@som.umaryland.edu (J.A.S.); mitchell.moyer@som.umaryland.edu (M.M.); ntsymbalyuk@som.umaryland.edu (N.T.); otsymbalyuk@som.umaryland.edu (O.T.); kkeledjian@som.umaryland.edu (K.K.); sivanova@som.umaryland.edu (S.I.); ctosun@som.umaryland.edu (C.T.); vgerzanich@som.umaryland.edu (V.G.); 2 Department of Pathology, University of Maryland School of Medicine, Baltimore, MD 21201, USA; 3 Department of Physiology, University of Maryland School of Medicine, Baltimore, MD 21201, USA

**Keywords:** cerebral ischemia, stroke, brain swelling, diabetes, astrocyte, SGLT2, canagliflozin

## Abstract

Brain swelling is a major cause of death and disability in ischemic stroke. Drugs of the gliflozin class, which target the Na^+^-coupled D-glucose cotransporter, SGLT2, are approved for type 2 diabetes mellitus (T2DM) and may be beneficial in other conditions, but data in cerebral ischemia are limited. We studied murine models of cerebral ischemia with middle cerebral artery occlusion/reperfusion (MCAo/R). *Slc5a2*/SGLT2 mRNA and protein were upregulated de novo in astrocytes. Live cell imaging of brain slices from mice following MCAo/R showed that astrocytes responded to modest increases in D-glucose by increasing intracellular Na^+^ and cell volume (cytotoxic edema), both of which were inhibited by the SGLT2 inhibitor, canagliflozin. The effect of canagliflozin was studied in three mouse models of stroke: non-diabetic and T2DM mice with a moderate ischemic insult (MCAo/R, 1/24 h) and non-diabetic mice with a severe ischemic insult (MCAo/R, 2/24 h). Canagliflozin reduced infarct volumes in models with moderate but not severe ischemic insults. However, canagliflozin significantly reduced hemispheric swelling and improved neurological function in all models tested. The ability of canagliflozin to reduce brain swelling regardless of an effect on infarct size has important translational implications, especially in large ischemic strokes.

## 1. Introduction

Acute ischemic stroke is a leading cause of death and disability worldwide [1]. In ischemic stroke, morbidity and mortality are determined in part by secondary injury, the most important in the acute phase being brain swelling. In non-lethal cerebral infarction, brain swelling is an independent predictor of poor outcome [2], and in large hemispheric infarction, brain swelling places patients at high risk for neurological deterioration and is largely responsible for the high mortality rate of 50–80% [3]. The only proven treatment for severe brain swelling is decompressive craniectomy, a lifesaving but morbid surgical procedure that involves removing a large part of the cranium [4]. 

The energy demands of neurons and other cells in the normal brain are met by several glucose transporters, of which there are two major classes: the passive facilitative glucose transporters belonging to the *Slc2* family (GLUT1, GLUT2, GLUT3, and GLUT4) and the secondary active Na^+^-coupled D-glucose cotransporter belonging to the *Slc5a* family (SGLT1) [5]. In contrast to D-glucose transport via GLUT transporters, Na^+^/D-glucose transport via SGLT is an energy-consuming process that may worsen the energy deficit during brain ischemia [5], suggesting that the inhibition of SGLT may be beneficial in brain ischemia.

SGLT2 is abundantly expressed in the kidney, where it acts to reabsorb glucose from urine [6], and SGLT1 is constitutively expressed in the brain, where it has a role in cerebral ischemia [7,8,9,10,11]. By comparison, SGLT2 in the brain is poorly understood. SGLT2, encoded by *Slc5a2*, functions as a low-affinity, high-capacity transporter; it operates with a Na^+^/D-glucose stoichiometry of one; and it uses the electrochemical gradient of Na^+^ ions to drive the transport of D-glucose against its concentration gradient [6]. Since *Slc5a* family members cotransport Na^+^, the transport of D-glucose into the cell by members of this family is accompanied by the depolarization of the cell membrane potential [6]. Depolarization by SGLT may exacerbate other depolarizing influences, such as spreading depolarization, known to be harmful in brain ischemia [12,13], further suggesting that the inhibition of SGLT may be beneficial in brain ischemia.

Recent evidence supports the hypothesis that inhibiting SGLT2 may be beneficial in brain ischemia. A meta-analysis showed that patients with type 2 diabetes mellitus (T2DM) treated with one of the subtype selective SGLT2 inhibitors (gliflozin) exhibited significant protection in terms of cerebrovascular and cardiovascular outcomes and mortality compared to T2DM patients treated with other agents [14]. Preclinical studies in rodent models of acute ischemic stroke have implicated SGLT2, based on experiments with empagliflozin and luseogliflozin, which target SGLT2 over SGLT1 with selectivity ratios of 2500:1 and 1650:1, respectively [15,16]. In a global ischemia model, treatment with empagliflozin resulted in better neurological function and smaller infarct volumes in both non-diabetic and diabetic rats [17,18]. In a permanent middle cerebral artery occlusion model, two-week pre-treatment with luseogliflozin resulted in better neurological function and smaller infarct volumes in non-diabetic mice [19].

Here, we studied the expression and function of SGLT2 in murine acute ischemic stroke models with middle cerebral artery occlusion/reperfusion (MCAo/R). We studied both non-diabetic mice and mice with T2DM, since canagliflozin is commonly used to treat patients with T2DM [20], and we studied mouse models with different severities (durations) of ischemia, due to the greater susceptibility of diabetics to ischemia [21,22,23]. We report that *Slc5a2* mRNA and SGLT2 protein were upregulated de novo in astrocytes. Our data indicate that, in post-MCAo/R astrocytes, the inhibition of SGLT2 reduced Na^+^ influx and cell swelling induced by D-glucose, and in post-MCAo/R mice, the inhibition of SGLT2 invariably reduced brain swelling and improved neurological function and, depending on the severity of the ischemic insult, reduced infarct volume.

## 2. Materials and Methods

Ethics statement. The animal experiments comply with the ARRIVE guidelines and were performed under a protocol approved by the Institutional Animal Care and Use Committee of the University of Maryland School of Medicine, and in accordance with the relevant guidelines and regulations as stipulated in the United States National Institutes of Health Guide for the Care and Use of Laboratory Animals.

Subjects. Male mice (22–28 gm) were used for the experiments. Wild-type (WT) C57BL/6NHsd mice were obtained from Envigo (Indianapolis, IN, USA).

Some experiments utilized a mouse model of non-insulin-dependent T2DM (Figure S1). Insulin resistance was induced by feeding a high-fat diet (60% fat; cat #D12492; Research Diets Inc., New Brunswick, NJ, USA). On week 4, hyperglycemia was induced by injecting these mice with two doses, two days apart, of streptozotocin (100 mg/kg) plus nicotinamide (240 mg/kg), for the partial destruction of pancreatic β cells leading, to a 50% reduction in pancreatic insulin [24,25].

For live cell imaging, mice with the astrocyte-specific expression of tdTomato were obtained by crossing ROSA26-tdTomato mice (B6.Cg-*Gt(ROSA)26Sor*^tm14(CAG-tdTomato)Hze^/J; cat. #007914; Jackson Laboratories, Bar Harbor, ME, USA) with GFAP-Cre mice (B6.Cg-Tg(Gfap-cre)73.12Mvs/J; cat. #012886; Jackson Laboratories). 

The *Slc5a2*^fl/fl^ mouse (loxP sites inserted to flank exons 4 and 5) was developed with a commercial vendor (Taconic Biosciences, Germantown, NY, USA) using CRISPR/Cas9-mediated genome engineering. The validation of the *Slc5a2*^fl/fl^ mouse is shown (Figure S2). Mice with the astrocyte-specific deletion of *Slc5a2* (Ast-*Slc5a2*^KO^ mice) were obtained by crossing *Slc5a2*^fl/fl^ mice with *Gfap* promoter-driven Cre/ERT2 mice (B6.Cg-Tg(Gfap-cre/ERT2)505Fmv/J; stock number 012849; Jackson Laboratories). Four–six weeks before the experiments, these mice were injected with AAV5-gfaABC_1_D-tdTomato (cat. #44332, Addgene, Watertown, MA, USA) to express the fluorescent reporter tdTomato in astrocytes. Two–three weeks prior to experiments, *Slc5a2*/SGLT2 deletion was induced by administering tamoxifen (2 mg in 100 μL of corn oil IP daily ×5 days) [26].

**Figure 1:**
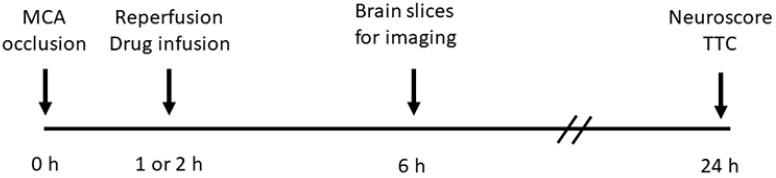
Schematic of experimental timeline.

Middle cerebral artery occlusion/reperfusion (MCAo/R). Under general anesthesia (induction, 3.0% isoflurane; maintenance, 1.5–2.0% isoflurane with a 20%/80% mixture of O_2_/N_2_O), MCAo was induced as described [27] using a silicon filament occluder (602356PK5Re Doccol Corp, Sharon, MA, USA) for 1 h or 2 h of occlusion followed by common carotid artery ligation. Relative cerebral blood flow, measured via laser Doppler flowmetry (LDF) (DRT4, Moor Instruments, Axminster, Devon, UK), was reduced 65% or more to (mean ± S.D.) 77.4 ± 5.8%. Mice were euthanized at 6 or 24 h according to the timeline in Figure 1 and as detailed below. Prespecified exclusion criteria included (1) LDF reduction < 65%; (2) absence of circling behavior after emerging from anesthesia; (3) subarachnoid hemorrhage identified at necropsy (incidence, 2–3%).

Following MCAo/R, the mice were used as follows: Series 1: Non-diabetic WT mice with MCAo/R (2/6 h) were used for immunohistochemistry (6 mice), RNAScope (3 mice), and the isolation of astrocytes for qPCR and immunoblot (3 mice); 3 uninjured control mice were used for the astrocyte isolation for qPCR and immunoblot. Series 2: Non-diabetic wild-type mice (12 mice), Ast-*Slc5a2*^KO^ (3 mice), and littermate controls (Ast-*Slc5a2*^WT^) (3 mice), all with astrocyte expression of tdTomato, underwent MCAo/R (2/6 h) and were used for the live cell imaging of Na^+^ and cell volume of astrocytes in brain slices. Series 3: T2DM mice with MCAo/R (1/24 h) were treated with canagliflozin vs. vehicle (13 vs. 10 mice) and used for measurements of stroke outcomes. Series 4: Non-diabetic mice with MCAo/R (1/24 h) were treated with canagliflozin vs. vehicle (16 vs. 11 mice) and used for measurements of stroke outcomes. Series 5: Non-diabetic mice with MCAo/R (2/24 h) were treated with canagliflozin vs. vehicle (21 vs. 35 mice) and used for measurements of stroke outcomes; additional mice treated with canagliflozin vs. vehicle (11 and 10 mice) were used to measure brain water. For series 5 only, in addition to the exclusion criteria listed above, additional prespecified exclusion criteria were death prior to 24 h of reperfusion and infarct volumes < 50 mm^3^. The last criterion resulted in uniformly large infarcts across groups, regardless of treatment, and allowed the study of brain swelling independent of infarct volume [28].

Treatment. At the time of reperfusion, the external jugular vein was exposed, and mice were randomly assigned to receive 200 μL of vehicle (0.6% DMSO in saline containing 1% (2-hydroxylpropyl)-γcyclodextrin) intravenously (IV) or 200 μL of vehicle containing canagliflozin (5.34 μg; ~200 μg/kg) IV. The dose of canagliflozin (5.34 μg/mouse) was based on that used in diabetic humans, typically 300 mg *per os*, which yields a measured therapeutic plasma level of 2.5 μg/mL [29]. For a 25 gm mouse with a blood volume of 80 μL/gm, a dose of 5.34 μg IV gives a calculated plasma level of 2.67 μg/mL (6.0 μM), similar to plasma levels in humans. With a brain/serum ratio = 0.3 [30], this dose gives a calculated brain concentration of 1.8 μM. Because this concentration is higher than the IC_50_ (2 nM for canagliflozin at mouse SGLT2 [31]), no adjustment for mouse body weight was made in the dose administered. The scientist performing the surgery and drug administration was “masked” to the treatment group.

Blood glucose was measured at reperfusion and at euthanasia using a glucometer.

Neurological function was evaluated by a “masked” investigator using the modified Garcia scoring system [32].

Infarct volume, hemispheric swelling, and excess water [27]. After euthanasia, brains were harvested, and 2 mm coronal sections were prepared using a chilled brain slicer matrix (Ted Pella, Inc., Redding, CA, USA). Brain slices were immersed in 2,3,5-triphenyltetrazolium chloride (TTC) at 22 °C × 10 min. Stained sections were imaged at 600 dpi using a flatbed scanner. Images were processed using the National Institutes of Health Image J software 1.52a with an open-source, semi-automated “plug-in”, which featured automatic-thresholding that yields reliable, unbiased measurements of TTC-negative and hemisphere areas [33]. Infarct volume (mm^3^) was calculated by multiplying the software-determined TTC-negative infarct area (mm^2^) by the slice thickness (2 mm) and summing infarct volumes across slices. To correct for tissue swelling, the total infarct volume was divided by the swelling factor, calculated as the ipsilateral hemisphere area/contralateral hemisphere area. Hemispheric swelling was calculated as (ipsilateral hemisphere volume/contralateral hemisphere volume)—1, expressed as percent. In separate experiments, excess water was measured using the wet weight/dry weight method, 100 × (W_W_ − W_D_)/W_W_, where W_W_ is the wet weight and W_D_ is the dry weight [34], using a Mettler Toledo Ab54-S Analytical Balance (Columbus, OH, USA).

Anti-SGLT2 antibody validation. We studied SGLT2 expression using a commercial rabbit polyclonal anti-SGLT2 antibody (#NBP1-92384; Novus Biologicals, Centennial, CO, USA). This antibody was independently validated previously [35], and here, we performed further validation to confirm its specificity. Mouse kidney and lung tissues, which have the greatest and least *Slc5a2*/SGLT2 expression, respectively [6], were used as positive and negative controls. Additional controls included choroid plexus [35] and COS-7 cells transiently transfected with plasmids containing cyan fluorescent protein (CFP)-tagged hSGLT1 and hSGLT2 using Lipofectamine 2000 per the manufacturer’s instructions (cat. #11668019; ThermoFisher, Scientific, Waltham, MA, USA) (CFP-tagged *Slc5a1* and *Slc5a2* plasmids were generously provided by Drs. Bernard Ribalet and Scott John, David Geffen School of Medicine, UCLA) [36]. The immunoblot of the kidney lysate showed a pair of bands, ~60 and ~65 kDa, with a third band, ~26 kDa, which were absent or minimal in lung lysate and were blocked by pre-absorption of the anti-SGLT2 antibody with the recombinant SGLT2 antigen peptide (#NBP1-92384PEP; Novus Biologicals) (Figure S3a). Previous reports on the immunoblot of mouse kidney and HEK-293T cells overexpressing *Slc5a2*/SGLT2 showed similar patterns of bands with principal bands at ~55–72 and ~26 kDa for SGLT2 [19,35]. Following the expression of CFP-tagged SGLT1 and SGLT2 in COS-7 cells, the immunoblot identified the SGLT2 expression system, not the SGLT1 expression system, with the SGLT2 signal blocked by pre-absorption with the recombinant SGLT2 antigen peptide (Figure S3b). The immunohistochemistry of the kidney tissue and choroid plexus showed the labeling of cells known to express SGLT2, which was blocked by pre-absorption with the recombinant SGLT2 antigen peptide (Figure S3c,e). Additional confirmation of the specificity of the Novus antibody was obtained by double-labeling kidney sections for *Slc5a2*/SGLT2 mRNA and protein. Immunohistochemistry with anti-SGLT2 antibody followed by RNAScope for *Slc5a2* showed extensive co-labeling with minimal protein labeling in areas without mRNA labeling (Figure S3d).

Immunohistochemistry. Under deep anesthesia, mice were euthanized and underwent transcardiac perfusion with normal saline (NS) (20 mL), followed by 10% neutral buffered formalin (20 mL) or 4% paraformaldehyde (20 mL). Brains were harvested and post-fixed. Tissues were cryoprotected with 30% sucrose, frozen in OCT, and cryosectioned (10 µm). Immunohistochemistry was performed as we described [28,37] in a non-blinded manner. In some cases, sections were first processed for antigen retrieval in Epitope Retrieval Solution (cat# IW-1100; IHC World, Ellicott City, MD, USA) using an Epitope Retrieval Steamer (cat# IW-1102; IHC World) for 10–15 min, followed by cooling for 30 min, then washing in distilled H_2_O. For all immunolabelings, sections were incubated at 4 °C overnight with primary antibodies directed against SGLT2 (1:50; #NBPI-92384; Novus Biologicals), NeuN (1:100; #ABN90P; MilliporeSigma, Burlington, MA, USA), or GFAP (1:200; #C9205, MilliporeSigma). After several rinses in PBS, sections were incubated with species-appropriate fluorescent secondary antibodies (Alexa Fluor 488 and 555, Molecular Probes, ThermoFisher Scientific, Waltham, MA, USA) for 1 h at room temperature. Controls for immunohistochemistry included the omission of primary antibodies. Unbiased assessments of specific labeling were obtained using NIS-Elements AR software (v.5.30, Nikon Instruments, Melville, NY, USA) from sections (one section per mouse) immunolabeled as a single batch. All images for a given signal were captured using uniform parameters of magnification, area, exposure, and gain.

Immunoblot for SGLT2 was performed as we described [28,38].

qPCR for *Slc5a2* was performed as we described [38,39] using the following primers: (5’ to 3′: forward CAGACCTTCGTCATTCTTGCCG; reverse GTGCTGGAGATGTTGCCAACAG). Relative C_T_ values were normalized to *Hprt* as the housekeeping gene (5′ to 3′: forward CTGGTGAAAAGGACCTCTCGAAG; reverse CCAGTTTCACTAATGACACAAACG).

RNAScope was performed using a commercial kit (Multiplex Fluorescent Detection v2 kit, Advanced Cell Diagnostics, Newark, CA, USA) according to the manufacturer’s protocol, using the following probes: mouse Aqp4 (cat #417161) and mouse Slc5a2 (cat #498131-C2).

Astrocyte isolation was performed using the Miltenyi Biotec adult mouse astrocyte isolation protocol (cat. 130-097-678, Miltenyi Biotec, Gaithersburg, MD, USA), as we described [38]. For post-MCAo/R experiments, astrocytes were isolated from ipsilateral MCA tissues. 

Live cell imaging of astrocytes in ex vivo brain slices was performed using mice with astrocyte expression of tdTomato (ROSA26-tdTomato;+GFAP-Cre mice) following MCAo/R (2/6 h). Mice were injected intraperitoneally (IP) with a lethal dose of sodium pentobarbital then exsanguinated via the transcardiac perfusion of ice-cold normal saline. The brains were rapidly extracted and transferred to a carbogen (95% O_2_/5% CO_2_)-saturated, ice-cold slicing solution containing (in mM) 222.1 sucrose, 27 NaHCO_3_, 1.4 NaH_2_PO_4_, 2.5 KCl, 0.5 ascorbic acid, 1 CaCl_2_ and 7 MgSO_4_. Coronal slices (300 µm) were prepared using a VT1200S vibratome (Leica Biosystems, Wetzlar, Germany). Slices from +1.0 to −2.0 mm relative to the bregma were sectioned at the midline and hemislices ipsilateral to MCAo/R were transferred to carbogen-saturated recovery artificial cerebrospinal fluid (aCSF) containing (in mM) 119 NaCl, 27 NaHCO_3_, 1.0 NaH_2_PO_4_, 2.5 KCl, 2 D-glucose, 8 mannitol, 2.5 CaCl_2_ and 1.3 MgCl_2_. Hemislices in recovery aCSF were gradually recovered to room temperature then to experimental conditions at 31–33 °C for at least 30 min prior to live cell imaging. A single 500 µm thick coronal slice at approximately the bregma zero coordinate was separately immersed in 1.5% TTC for 10 min at room temperature to confirm the ischemic injury. Imaging experiments were not conducted if a grossly visible infarct area was not identified. 

For Na^+^ imaging, 50 µg of the AM-ester form of ING-2 [40] (Ion Biosciences, San Marcos, TX, USA) was dissolved in 8 µL of DMSO and 2 µL of pluronic F-127 solution (20% in DMSO). Ex vivo hemislices were surface loaded with the ING2-AM dye mix in 10 mL of aCSF for 30 min at room temperature, then transferred to the recording chamber where the hemislice was further recovered in carbogen-saturated aCSF superfusion conditions. The composition of baseline experimental aCSF for Na^+^ imaging was the same as the recovery aCSF but supplemented with 20 mM mannitol. The superfusion of aCSF in the recording chamber was maintained at a flow rate of 5 mL/min with a chamber exchange time of 27 ± 1 s. Intensity-based Na^+^ imaging was performed using the spinning disk confocal microscope (CSU-W1 Nikon, Melville, NY, USA) equipped with a 20×/0.75 NA objective and further resolved via a live-SR super-resolution imaging module. Fluorescence signals of ING2 (ex/em 525 nm/545 nm) and tdTomato (excitation/emission, 561 nm/605 nm) were detected every 5 s. Baseline fluorescence in superfusion conditions were recorded for at least 5 min prior to introducing experimental conditions to the hemislice specimen. SGLT2 was activated by switching from aCSF containing 2 mM D-glucose to aCSF containing 10 mM D-glucose, with compensatory changes in mannitol concentration. Image processing and analysis were performed on the NIS Elements software (v.5.30, Nikon). Squared ROIs were drawn over single astrocyte somata as defined by the tdTomato fluorescence signal. Time-lapse fluorescence values were acquired from individual ROIs. Averaged transients (ΔF/F_0_) were calculated and expressed as mean ± S.E.

For astrocyte cell volume imaging, Z-stack images of tdTomato-positive astrocytes were acquired at 5 min intervals during a 30 min protocol. Images were acquired in baseline aCSF containing 2 mM D-glucose for 2.5 min. SGLT2 was activated by switching to aCSF containing 10 mM D-glucose for the remainder of the experiment. Images were processed using the NIH ImageJ software (v. 1.52a) to correct for x-y drift and fluorescence fading [41]. The areas of individual astrocytes for each Z-plane were used to determine cell volume.

Data analysis. Data are presented as mean ± S.E. unless otherwise noted. Student’s *t*-test, 1-way ANOVA with Bonferroni post hoc comparisons, or the Mann–Whitney U test were used, as appropriate. Analyses were performed with Origin Pro V8 or GraphPad Prism 8.3. *p* < 0.05 was deemed to be statistically significant.

## 3. Results 

### 3.1. SGLT2 Expression

**Figure 2:**
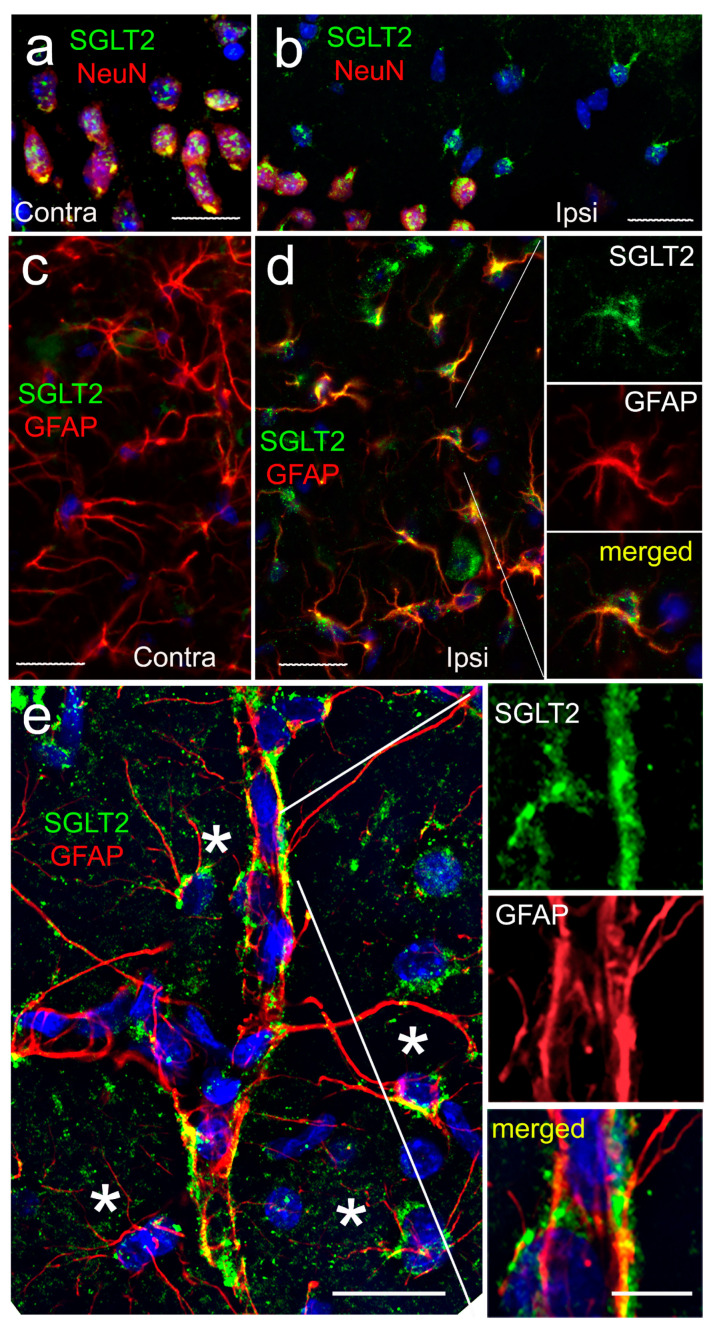
SGLT2 is upregulated in astrocyte cell bodies and astrocyte endfeet following cerebral ischemia. (**a**–**e**) Double immunolabeling for SGLT2 (green) and NeuN (**a**,**b**) or GFAP (**c**–**e**) (red) in contralateral (Contra) and ipsilateral (Ipsi) brain sections from mice post-MCAo/R (2/6 h); merged images are shown in a, b, d, e; individual labelings are shown in c, d—inset, e—inset; the images shown are representative of findings in 3 mice; scale bars, 25 μm except insert, 10 μm.

Immunolabeling for SGLT2 was performed on brain sections from mice following MCAo/R (2/6 h). Sections were immunolabeled with an anti-SGLT2 antibody that we validated independently (Figure S3), and they were co-immunolabeled to identify cell-specific expression. SGLT2 was identified in NeuN-positive neurons, both ipsilateral and contralateral to MCAo/R, with no apparent difference due to ischemia (Figure 2a,b). The similar constitutive expression of SGLT2 in neurons was observed in uninjured control mice (not shown) [42].

By contrast, astrocytes showed the robust de novo upregulation of SGLT2. In GFAP-positive astrocyte cell bodies, SGLT2 expression was minimal in contralateral tissues but was prominent ipsilateral to MCAo/R (Figure 2c,d). In addition, SGLT2 expression was prominent in GFAP-positive perivascular structures consistent with perivascular astrocyte endfeet (Figure 2e).

**Figure 3:**
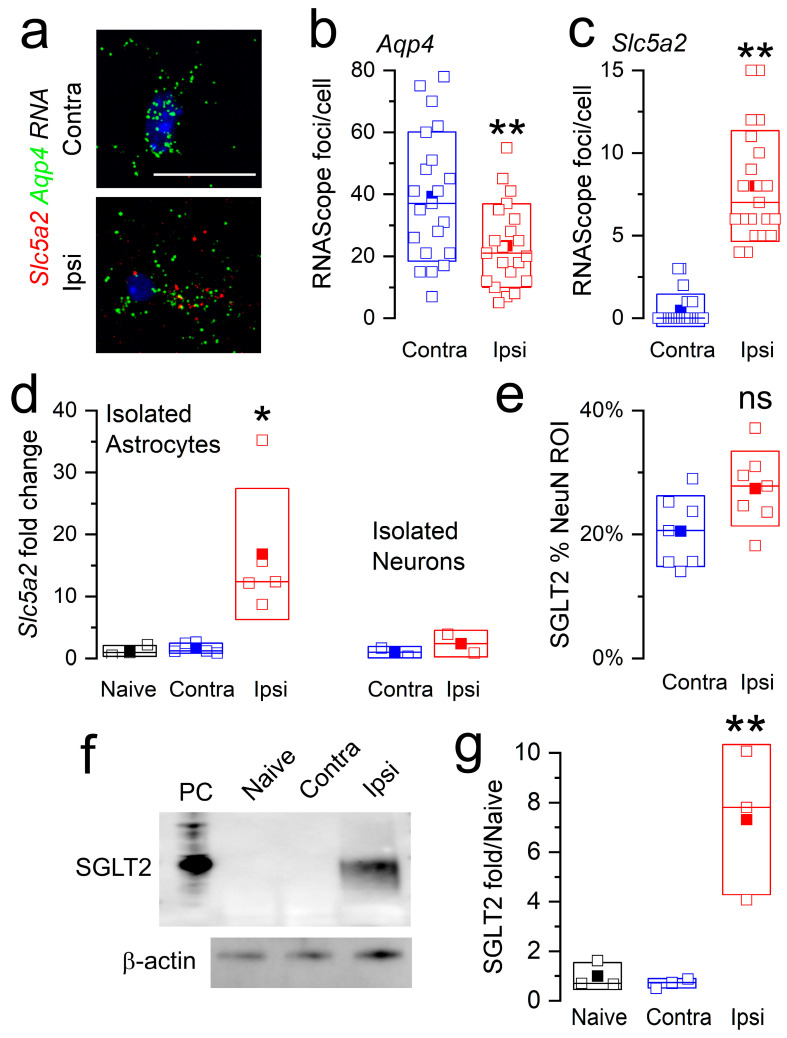
*Slc5a2* mRNA and SGLT2 protein are upregulated in astrocytes following cerebral ischemia. (**a**–**c**) Images (**a**) and quantification (**b**,**c**) of RNAscope for *Slc5a2* (red) and *Aqp4* (green) mRNA in contralateral (Contra) and ipsilateral (Ipsi) brain sections from mice post-MCAo/R (2/6 h); scale bar, 25 μm; data from 3 mice per group; **, *p* < 0.01. (**d**) qPCR for *Slc5a2* mRNA in astrocytes and neurons isolated from the contralateral (Contra) and ipsilateral (Ipsi) MCA territory of mice following MCAo/R (2/6 h) and uninjured controls (naïve), expressed as fold-change; 5 mice per group; *, *p* < 0.05. (**e**) Quantitative immunohistochemistry for SGLT2 expressed within regions of interest determined by NeuN; data from 7 mice per group; ns, not significant. (**f**,**g**) Immunoblot (**f**) and quantification of immunoblot (**g**) for SGLT2 in astrocytes isolated from the contralateral (Contra) and ipsilateral (Ipsi) MCA territory of mice following MCAo/R (2/6 h) and uninjured controls (naïve), expressed as fold-change; PC, positive control (kidney lysate); β-actin used as a loading control; 3 mice per group.

The upregulation of SGLT2 in astrocytes was corroborated in tissue sections using RNAScope for *Slc5a2* and *Aqp4* (aquaporin 4) mRNA (Figure 3a). Ipsilateral tissues showed a slightly reduced number of perinuclear foci for *Aqp4* mRNA (Figure 3b) [43]. However, the number of foci for *Slc5a2* mRNA was significantly increased in *Aqp4*-positive cells (Figure 3c). The findings with RNAScope corroborated qPCR on cells isolated from the MCA territory following MCAo/R (2/6 h). Astrocytes isolated from ipsilateral MCA tissues showed a 12-fold increase in *Slc5a2* mRNA, compared to minimal changes in astrocytes from the contralateral MCA tissues or from uninjured animals (Figure 3d). The qPCR of isolated neurons showed no change in *Slc5a2* mRNA (Figure 3d). 

SGLT2 protein expression mirrored *Slc5a2* mRNA expression. Quantitative immunohistochemistry showed no significant change in SGLT2 expression in NeuN-positive neurons (Figure 3e). By contrast, the immunoblot of astrocytes isolated from the MCA territory following MCAo/R (2/6 h) showed minimal SGLT2 protein in contralateral astrocytes but robust expression in ipsilateral astrocytes (Figure 3f,g), mirroring the findings from immunohistochemistry (Figure 2c,d).

### 3.2. Live Cell Imaging of Astrocytes in Post-MCAo/R Brain Slices

To study the function of newly upregulated SGLT2 in astrocytes, we performed MCAo/R (2/6 h) in ROSA26-tdTomato;+GFAP-Cre mice, which express the fluorescent protein, tdTomato, in astrocytes. Tissues were studied 8 h or more after the onset of ischemia with the goal of elucidating early events that would predispose to brain swelling. Ex vivo brain slices from these mice were used for the live cell imaging of astrocytes. SGLT2 was activated by exposing brain slices to a modest glycemic stimulus, consisting of a step-change from 2 to 10 mM D-glucose, with a compensatory adjustment in osmolarity using mannitol.

#### 3.2.1. Na^+^ Imaging

**Figure 4:**
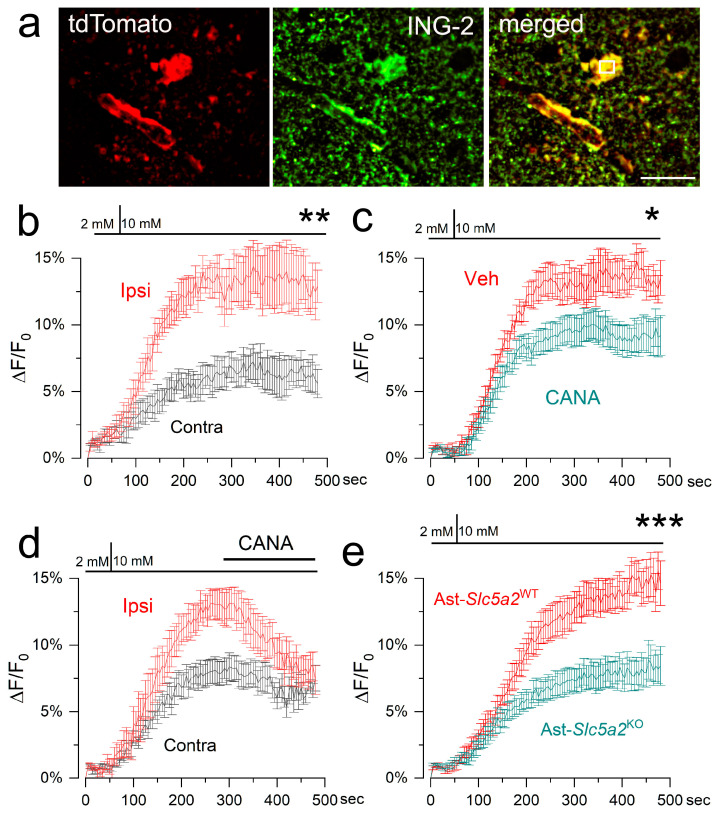
D-glucose-induced Na^+^ influx in post-ischemic astrocytes is inhibited by canagliflozin and by the astrocyte-specific deletion of *Slc5a2*/SGLT2. (**a**) Live cell image of a tdTomato-expressing astrocyte in an ex vivo brain slice from an MCAo/R (2/6 h) mouse, following incubation with ING-2; scale bar, 25 μm. (**b**–**e**) Time course of Na^+^ influx with step change in D-glucose from 2 to 10 mM in ipsilateral (Ipsi) vs. contralateral (Contra) astrocytes (**b**); in ipsilateral astrocytes without and with pre-incubation with canagliflozin (5 μM) (**c**); in ipsilateral astrocytes without and with the addition of canagliflozin (5 μM) at the time indicated by the bar (**d**); in ipsilateral astrocytes from Ast-*Slc5a2*^WT^ vs. Ast-*Slc5a2*^KO^ mice (**e**); * *p* < 0.05; ** *p* < 0.01; *** *p* < 0.001; 3–5 cells from 3 mice for each condition.

Na^+^ imaging showed that a step change in D-glucose gave rise to an increase in intracellular Na^+^ that was significantly larger in astrocytes from ipsilateral compared to contralateral brain slices (Figure 4a,b), consistent with the constitutive as well as de novo upregulation of SGLT in astrocytes. Preincubation with canagliflozin before the step change or the addition of canagliflozin during the glycemic challenge reduced the Na^+^ influx induced by D-glucose in ipsilateral astrocytes (Figure 4c,d), consistent with the involvement of SGLT2.

We also studied brain slices from mice with the astrocyte-specific deletion of *Slc5a2*/SGLT2 (Ast-*Slc5a2*^KO^) and littermate controls (Ast-*Slc5a2*^WT^) (Figure S2). In ipsilateral brain slices, a step change in D-glucose gave rise to an increase in intracellular Na^+^ that was significantly larger in astrocytes from Ast-*Slc5a2*^WT^ compared to Ast-*Slc5a2*^KO^ mice (Figure 4e), consistent with the involvement of SGLT2 in astrocytes.

#### 3.2.2. Cell Swelling

**Figure 5:**
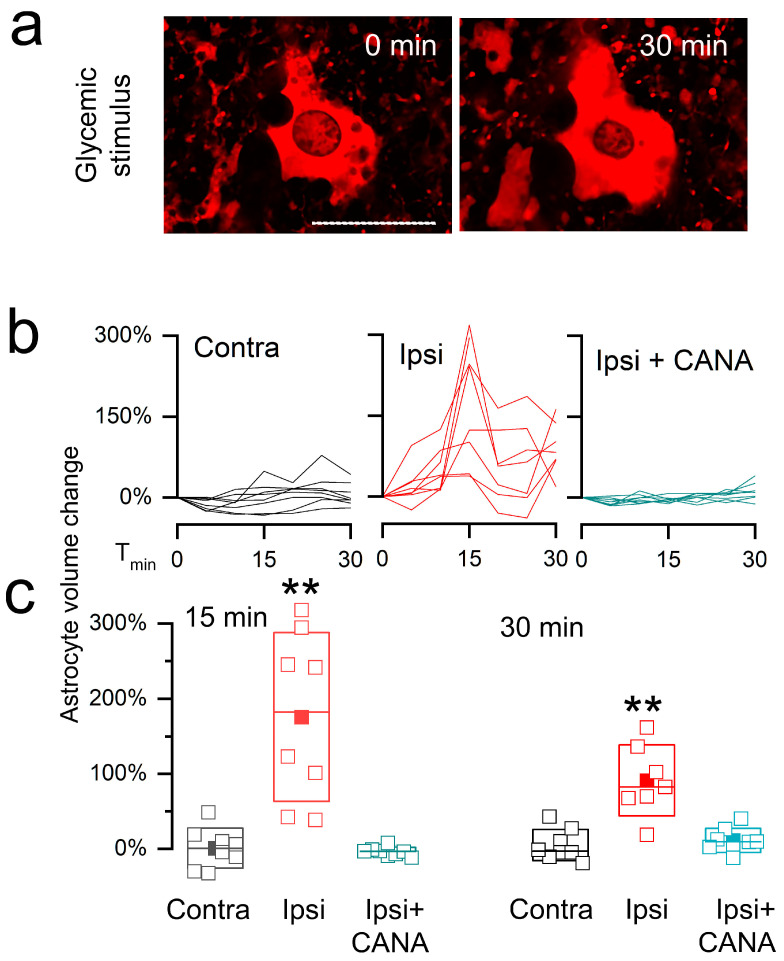
D-glucose-induced cell swelling of post-ischemic astrocytes is blocked by canagliflozin. (**a**) Live cell image of a tdTomato-expressing astrocyte in an ex vivo brain slice from an MCAo/R (2/6 h) mouse at baseline (0 min) and 30 min after a step change in D-glucose from 2 to 10 mM; bar, 25 μm. (**b**,**c**) Astrocyte volume changes in individual cells (**b**) and average changes (mean ± S.E.) (**c**) in astrocytes from contralateral brain (Contra), ipsilateral brain (Ipsi), and ipsilateral brain treated with canagliflozin (Ipsi + CANA; 5 μM); **, *p* < 0.01; 8 cells from 3 mice for each condition.

In slices from the contralateral brain, astrocytes volumes were minimally affected by the glycemic challenge (Figure 5). By contrast, in slices from the ipsilateral brain, astrocytes exhibited a marked phasic increase in volume that subsided only partially, resulting in a persistent increase in volume that was significantly greater than in controls (Figure 5a–c). Canagliflozin completely blocked the D-glucose-induced changes in volume in astrocytes from the ipsilateral hemisphere (Figure 5b,c), consistent with the dominant involvement of SGLT2 in the D-glucose-induced swelling response in astrocytes ipsilateral to the ischemic insult.

Apart from astrocyte cell swelling, the glycemic challenge also induced nuclear shrinkage in ipsilateral astrocytes (Figure 5a). Nuclear shrinkage accompanied by asymmetrical shape changes is typical of an osmotic perturbation [44].

### 3.3. Canagliflozin in MCAo/R Models

#### 3.3.1. MCAo/R (1/24 h) in T2DM Mice

We studied the effect of canagliflozin in cerebral ischemia in a mouse model of non-insulin-dependent T2DM induced by a high-fat diet plus streptozotocin [24,25]. This model mimics the metabolic characteristics of humans with T2DM associated with a Western diet [45,46,47,48]. In the mice, the high-fat diet was associated with weight gain, and animals exhibited hyperglycemia and glycosuria (Figure S1).

**Figure 6:**
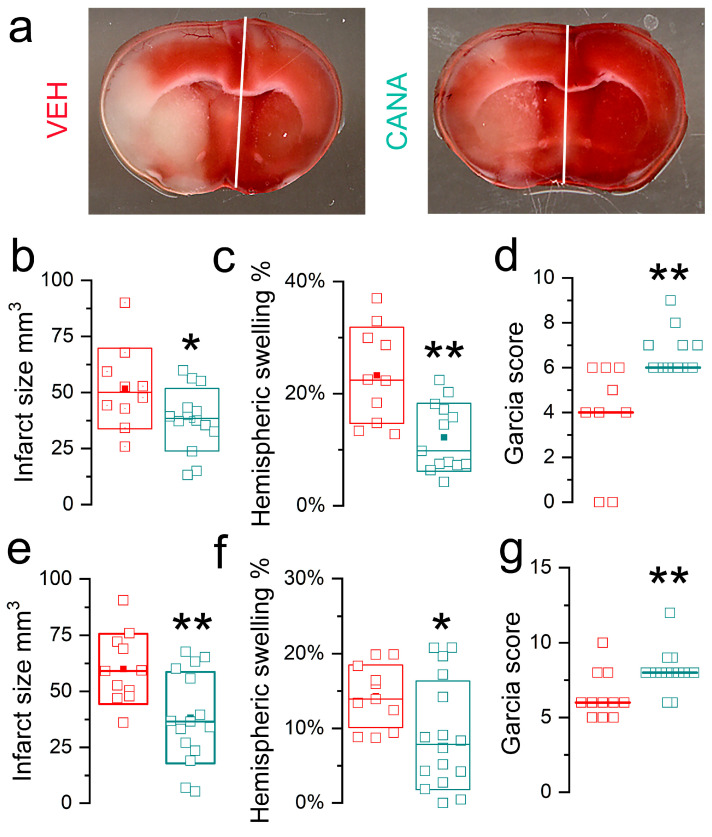
Effects of canagliflozin in mice with a moderate ischemic insult (MCAo/R, 1/24 h). (**a**) Images of TTC (2,3,5-triphenyltetrazolium chloride)-stained coronal sections from mice with type 2 diabetes mellitus (T2DM) administered vehicle (VEH) vs. canagliflozin (CANA) at reperfusion. (**b**–**d**) Infarct size (**b**), hemispheric swelling (**c**), and Garcia scores (**d**) for T2DM mice administered vehicle (red) vs. canagliflozin (green); Garcia score of 0 denotes death; 13 vs. 10 mice for CANA vs. VEH; *, *p* < 0.05; **, *p* < 0.01. (**e**–**g**) Infarct size (**e**), hemispheric swelling (**f**), and Garcia scores (**g**) for non-diabetic mice administered vehicle (red) vs. canagliflozin (green); 16 vs. 11 mice for CANA vs. VEH.

Cerebral ischemia was induced by MCAo/R (1/24 h), and at the time of reperfusion, mice were administered a single dose of canagliflozin (5.34 μg/mouse) or vehicle intravenously (IV). The dose of canagliflozin used here yielded calculated blood concentrations in the mice that mimicked the concentrations found in humans with T2DM who take canagliflozin (see Section 2). Infarct volumes were significantly reduced in canagliflozin vs. vehicle groups (37.8 ± 3.7 mm^3^ vs. 51.7 ± 5.7 mm^3^; *p* = 0.04) (Figure 6a,b). Drug treatment decreased hemispheric swelling by nearly half (12.2 ± 1.7% vs. 23.3 ± 2.7%; *p* = 0.002) (Figure 6c). Commensurate with these effects, drug treatment resulted in a significant improvement in neurological function (Garcia score medians: 6 vs. 4; *p* = 0.0007) (Figure 6d).

#### 3.3.2. MCAo/R (1/24 h) in Non-Diabetic Mice

For a comparison, we studied the effect of canagliflozin in non-diabetic mice following the same ischemic insult (MCAo/R, 1/24 h) and with the same dose of canagliflozin (5.34 μg/mouse IV at reperfusion). Infarct volumes were significantly reduced in canagliflozin vs. vehicle groups (38.3 ± 5.3 mm^3^ vs. 60.0 ± 4.7 mm^3^; *p* = 0.007) (Figure 6e), drug treatment decreased hemispheric swelling (9.1 ± 1.8% vs. 14.3 ± 1.3%; *p* = 0.04) (Figure 6f), and drug treatment resulted in a significant improvement in neurological function (Garcia score medians: 8 vs. 6; *p* = 0.003) (Figure 6g). Comparing data from vehicle-treated non-diabetic vs. vehicle-treated T2DM mice, infarct sizes were similar, but hemispheric swelling in T2DM mice (23.3 ± 2.7%) was more pronounced than in non-diabetic mice (14.3 ± 1.3%), consistent with known worsening of edema associated with hyperglycemia in cerebral ischemia/reperfusion [21,22,23].

#### 3.3.3. MCAo/R (2/24 h) in Non-Diabetic Mice

We studied the effect of canagliflozin in non-diabetic mice following a more severe ischemic insult (MCAo/R, 2/24 h). Similar to our previous report with this model [28], we only included mice with infarcts >50 mm^3^ to permit the study of brain swelling specifically in large infarcts. The same dose of canagliflozin as above (5.34 μg/mouse IV) was administered at the time of reperfusion.

**Figure 7:**
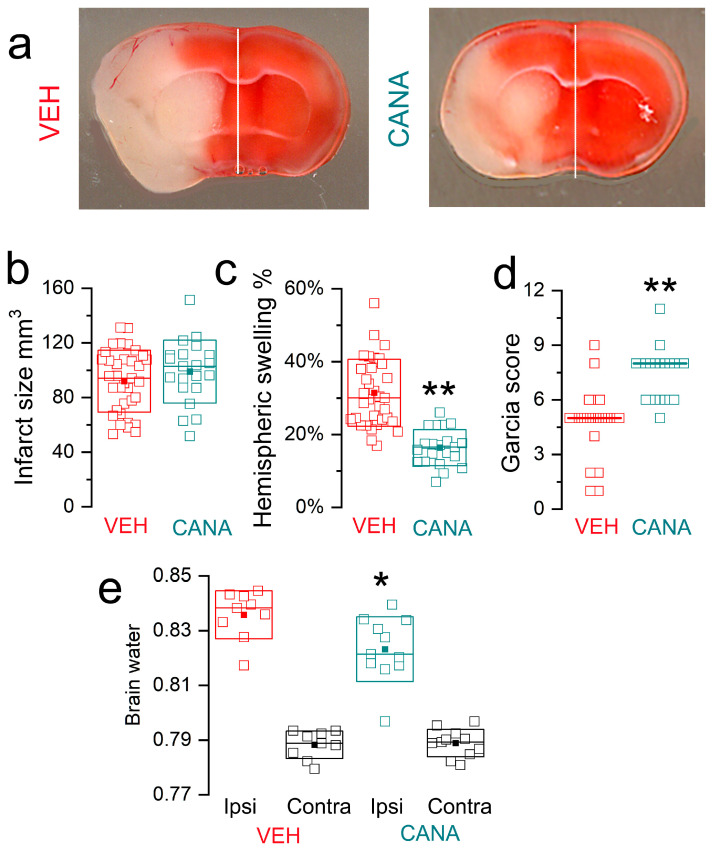
Effects of canagliflozin in mice with a severe ischemic insult (MCAo/R, 2/24 h). (**a**) Images of TTC (2,3,5-triphenyltetrazolium chloride)-stained coronal sections from non-diabetic mice administered vehicle (VEH) vs. canagliflozin (CANA) at reperfusion. (**b**–**d**) Infarct size (**b**), hemispheric swelling (**c**), and Garcia scores (**d**) for non-diabetic mice administered vehicle (red) vs. canagliflozin (green); 21 vs. 35 mice for CANA vs. VEH; ** *p* < 0.01. (**e**) Contralateral (Contra) and ipsilateral (Ipsi) brain water in non-diabetic mice administered vehicle vs. canagliflozin; 11 vs. 10 mice for CANA vs. VEH; * *p* < 0.05.

In this series, infarct volumes were not different in canagliflozin vs. vehicle groups (98.8 ± 5.0 mm^3^ vs. 91.9 ± 3.8 mm^3^; *p* = 0.27) (Figure 7a,b). However, similar to the finding in T2DM mice, drug treatment decreased hemispheric swelling by nearly half (16.4 ± 1.1% vs. 31.4 ± 1.7%; *p* = 5.5 × 10^−9^) (Figure 7c). The effect of the drug treatment on hemispheric swelling was corroborated in separate groups by measuring brain tissue water content, which was significantly reduced with the drug treatment (0.823 ± 0.004 vs. 0.836 ± 0.003; *p* = 0.016) (Figure 7e). The decrease in hemispheric swelling of 48% [(31 − 16)/31] was reflected in the decrease in excess brain water of 40% ([(0.84 − 0.79) − (0.82 − 0.79)]/[0.84 − 0.79]) [34]. 

Neurological function was better in the canagliflozin vs. vehicle groups (Garcia score medians: 8 vs. 5; *p* = 5.8 × 10^−9^) (Figure 7d), consistent with the observed reduction in brain swelling despite no difference in infarct size.

Blood glucose at reperfusion (175 ± 10 mg/dL vs. 166 ± 10 mg/dL; *p* = 0.6; 5–7 mice/group) and at 24 h (146 ± 10 mg/dL vs. 139 ± 14 mg/dL; *p* = 0.7; 5–7 mice/group) was not different between groups.

## 4. Discussion

Early preclinical studies in rodent models of stroke implicated SGLT, based on experiments with the non-selective agent, phlorizin [8,49,50], which has a selectivity ratio of 1.5:1 for SGLT2 over SGLT1 [51]. Here, we used the SGLT2-selective agent, canagliflozin, with a selectivity ratio of 250:1 for SGLT2 over SGLT1 [51]. We cannot exclude that some of the effects we observed with canagliflozin were not due to the inhibition of SGLT1, but the selectivity of canagliflozin strongly implicates the involvement of SGLT2. 

For our experiments with canagliflozin in a T2DM mouse model, we used a modest ischemic insult (MCAo/R, 1/24 h) due to the greater injury and lethality associated with more severe ischemia when combined with reperfusion [21]. With this model, we found that the drug improved neurological function and reduced infarct volumes and brain swelling. A similar but less robust effect of the drug on brain swelling was observed with the same ischemic insult in non-diabetic mice, underscoring the well-known harm that accompanies hyperglycemia in cerebral ischemia/reperfusion [21,22,23]. Our findings with canagliflozin align with reports that utilized other SGLT2-specific, gliflozin-type agents [17,18,19]. Overall, gliflozin treatment appears to be beneficial in various rodent models of moderate cerebral ischemia, with or without the complication of hyperglycemia. 

We also studied a mouse model with a severe ischemic insult known to be associated with marked brain swelling (MCAo/R, 2/24 h) [28]. In our experience with this model, brain swelling in untreated mice results in high mortality at 3–5 days, but mortality at 24 h is minimal, allowing the uncomplicated study of brain swelling at 24 h. In non-diabetic mice with MCAo/R (2/24 h), canagliflozin reduced brain swelling without reducing infarct volumes. The better neurological function with canagliflozin treatment, despite having no effect on infarct size, underscores the important benefit of reduced brain swelling on neurological function. The reduction in brain swelling with no effect on infarct volume observed here is similar to our recent report with the same model of severe ischemia, in which we evaluated multiple pharmacological treatments and gene deletions targeting SUR1-TRPM4 and NCX1 [28]. In that study, we emphasized the clinical importance of identifying molecularly directed treatments to dissociate brain swelling from infarct size, especially with large infarcts.

In addition to a non-salvageable infarct core, an ischemic insult produces an ischemic penumbra that may or may not be salvageable depending upon the promptness and adequacy of reperfusion. Most models of acute ischemic stroke incorporate a salvageable penumbra, and treatments found to reduce swelling or edema generally do so in proportion to the reduction in infarct size conferred by the treatment [52,53]. However, the effective clinical management of brain swelling requires molecularly informed treatments that act on edema regardless of infarct size, and without having to depend on a reduction in infarct volume to reduce swelling. The concept of reducing brain swelling independent of reducing infarct volume is clinically important, especially in large hemispheric infarctions [54]. Our data with the severe ischemia model suggest that canagliflozin may have translational potential in patients with large ischemic strokes at high risk of brain swelling.

At the dose used here in non-diabetic mice with a severe ischemic insult, we did not find any important effect of canagliflozin on blood glucose. Since the effect of canagliflozin on brain swelling was independent of blood glucose, and since canagliflozin readily penetrates the blood–brain barrier (brain/serum ratio = 0.3 [30]), the effect that we observed on brain swelling likely was mediated via SGLT2 expressed by brain cells, especially astrocytes.

SGLT1 was previously shown to be upregulated in neurons but not in astrocytes following cerebral ischemia [9]. Here, we found that, following MCAo/R, SGLT2 was expressed in neurons and de novo upregulated in astrocytes. We showed that, in post-MCAo astrocytes, a modest increase in extracellular D-glucose led to an increase in intracellular Na^+^ and astrocyte swelling, both of which were inhibited by canagliflozin. Our data indicated that astrocyte swelling was largely blocked by canagliflozin, whereas Na^+^ influx was reduced by only half or less by canagliflozin and by the astrocyte-specific deletion of *Slc5a2*/SGLT2. Since D-glucose-induced Na^+^ influx is not expected with GLUT transporters, these findings suggest that an SGLT subtype other than SGLT2 may be expressed in astrocytes, one that may contribute to cell volume in a manner that is different from SGLT2. Future work will be required to identify the mechanism of Na^+^ influx not attributable to SGLT2.

Our data indicate that SGLT2 is expressed constitutively in neurons and is de novo upregulated in astrocytes following cerebral ischemia. Previous reports showed the expression of SGLT2 protein in multiple regions of the normal brain, including the hypothalamus, amygdala, periaqueductal gray, and the nucleus of the solitary tract [42], as well as choroid plexus epithelial cells and ependymal cells [35]. SGLT2 also was identified in various diseases of the brain, including patients who died from traumatic brain injury [55] and microvessels in a mouse model of stroke [19]. Consistent with SGLT2 upregulation in cerebral ischemia, SGLT2 upregulation in brain pericytes was identified following oxygen–glucose deprivation, an in vitro model of stroke [19]. Further work will be required to identify the transcriptional mechanism responsible for *Slc5a2*/SGLT2 upregulation in astrocytes post-ischemia.

Certain limitations of this study should be acknowledged. We used canagliflozin for our studies, due to its better water solubility for intravenous administration compared to other gliflozins. Canagliflozin targets SGLT2 over SGLT1 with a selectivity ratio of 250:1, whereas other gliflozins have better selectivity for SGLT2 [51]. Future experiments with the cell-specific gene deletion of *Slc5a2* will be required confirm our findings with canagliflozin on brain swelling. The Na^+^/H^+^ exchanger 1 (NHE-1) has been described as a potential off-target of empagliflozin [56,57], and NHE1 in astrocytes is known to be an important contributor to brain swelling in cerebral ischemia [58]. However, there is no consensus on this effect with empagliflozin [59], and canagliflozin has not been implicated in this effect. Finally, we only used male mice for our studies. Sex differences in *Slc5a2* mRNA and SGLT2 protein expression have been reported in mouse kidney [60], and canagliflozin was found to have sex-specific neuroprotective effects in aged mice [61]. Future experiments will be required to determine whether gliflozins have a different effect on astrocyte swelling and brain swelling in females.

## 5. Conclusions

SGLT2 is increasingly recognized for its importance is various diseases. SGLT2 was originally targeted for its role in glucose resorption in the kidney in patients with T2DM. SGLT2 now is viewed as having a potentially important role in cardiac and renal function, dementia, autism spectrum disorders, and other conditions in non-diabetic patients, independent of serum glucose [62,63,64,65]. The data presented here and previously [66,67] indicate that SGLT2 inhibitors also have potentially important protective effects in acute ischemic stroke. Moreover, in stroke, our data on SGLT2 as well as previous data on SUR1-TRPM4 and NCX1 [28] indicate that post-ischemic brain swelling is regulated by druggable cellular/molecular mechanisms that are distinct from those that govern neuronal death. Treatments that reduce brain swelling result in better neurological function even without an effect on infarct size, underscoring the clinical importance of targeting mechanisms of brain swelling in cerebral ischemia.

## 6. Patents

J.M.S., V.G., and J.A.S. have filed US Provisional Patent Application Number 63/324,492, filed 28 March 2022, titled “Methods and Compositions for the Treatment of Stroke”.

## Data Availability

The data presented in this study are openly available in Open Science Framework (OSF), reference number DOI 10.17605/OSF.IO/PY34R.

## Supplementary Materials

The following supporting information can be downloaded at: https://www.mdpi.com/article/10.3390/cells12182221/s1, Figure S1. Murine model of type 2 diabetes mellitus (T2DM). Figure S2. Astrocyte-specific deletion of *Slc5a2*/SGLT2 (Ast-*Slc5a2*^KO^). Figure S3. Validation of Novus anti-SGLT2 antibody.
