# Supplementary material for: Canagliflozin, an Inhibitor of the Na+-Coupled D-Glucose Cotransporter, SGLT2, Inhibits Astrocyte Swelling and Brain Swelling in Cerebral Ischemia
Source: Cells. 2023 Sep 6;12(18):2221. doi: 10.3390/cells12182221 (PMC10527352; doi:10.3390/cells12182221)
Supplement: Supplementary file 1 [file cells-12-02221-s001.zip › cells-2587089-supplementary.pdf]

**SUPPLEMENTARY MATERIALS**

**Canagliflozin, an inhibitor of the Na+-coupled D-glucose cotransporter, SGLT2, inhibits astrocyte swelling and brain swelling in cerebral ischemia.**

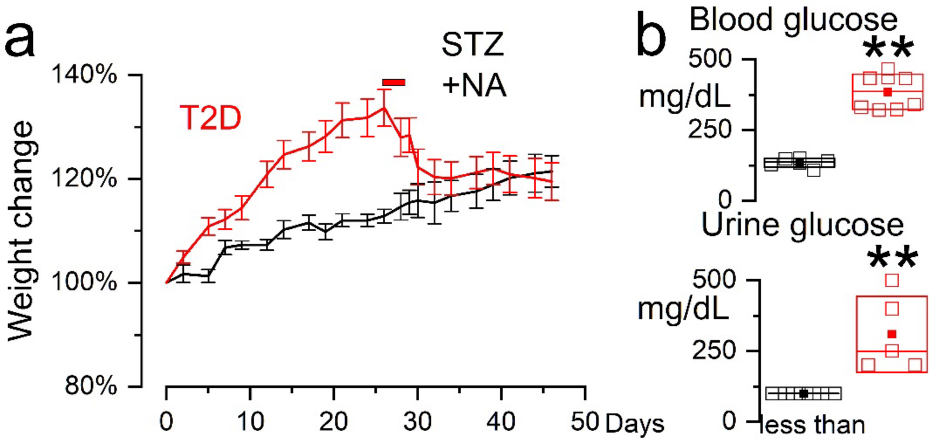


**Figure S1. Murine model of type 2 diabetes mellitus (T2DM).** (**a**) Change in weight of T2DM mice on high-fat diet (red) vs. control mice on normal diet (black); streptozotocin plus nicotinamide (STZ+NA) were administered at the time indicated by the bar; mean ± S.E.; 10–13 mice per group. (**b**) Blood and urine glucose in T2DM (red) vs. control mice (black) 46 days after starting the high-fat diet; 5–9 mice per group; $P<0.01$.

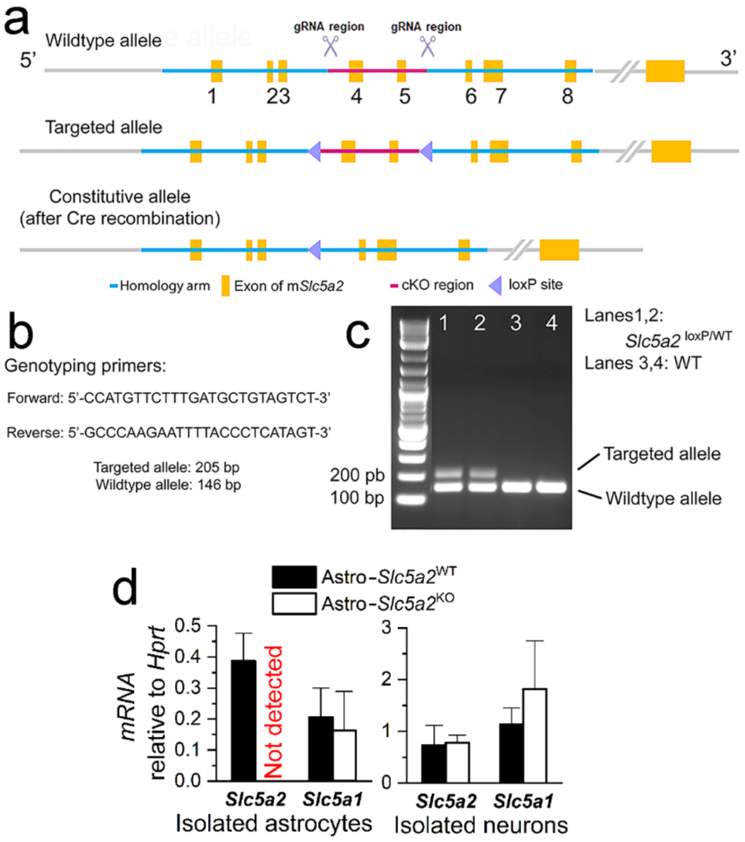


**Figure S2. Astrocyte-specific deletion of *Slc5a2*/SGLT2 (Ast-*Slc5a2*$^{KO}$). (a)** Schematic of targeted allele, and of the allele after Cre recombinase. **(b)** Genotyping primers used for validation. **(c)** PCR of DNA from heterozygous *Slc5a2*$^{loxP/WT}$ (lanes 1,2) and WT mice (lanes 3,4). **(d)** *Slc5a2* and *Slc5a1* mRNA in conditional *Slc5a2* knockout mice. *Slc5a2*$^{fl/fl}$;+*GFAP*-cre/ERT2 mice (Astro-*Slc5a2*$^{KO}$) and littermate controls (Astro-*Slc5a2*$^{WT}$) were administered tamoxifen and 2 weeks later underwent MCAo/R (2/6 hours). Isolated astrocytes and neurons from the ipsilateral MCA territory were analyzed by qPCR for *Slc5a2* and *Slc5a1* mRNA. Conditional knockout reduced *Slc5a2* in astrocytes (>40 cycles; "Not detected") but not in neurons and had no effect on *Slc5a1*; mean±SE; data from 3 mice.

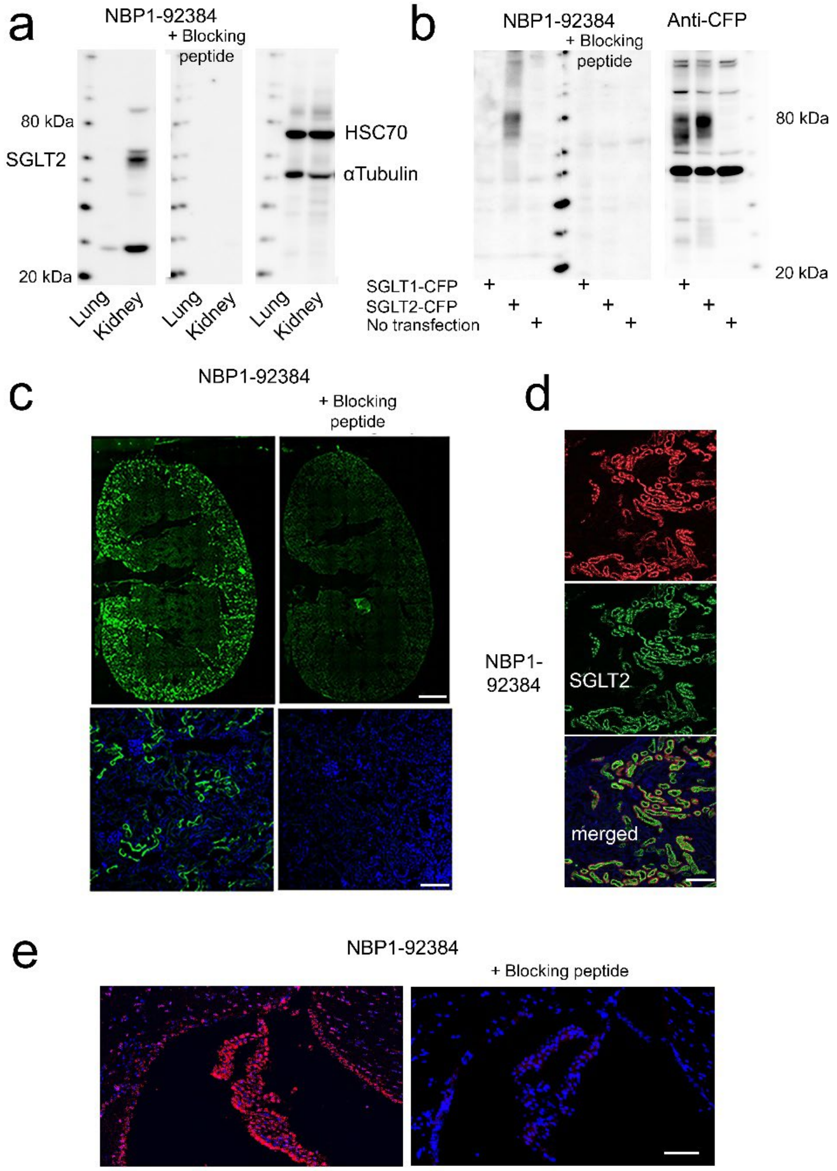


**Figure S3. Validation of Novus anti-SGLT2 antibody.** (**a**) Immunoblot of lysate from mouse kidney and lung tissues using Novus anti-SGLT2 antibody (#NBP1-92384; Novus Biologicals), without and with pre-absorption of the antibody with the recombinant SGLT2 antigen peptide (#NBP1-92384PEP; Novus Biologicals); immunoblot of HSC70 and α-tubulin were used as molecular weight and loading controls. (**b**) Following the expression of cyan fluorescent protein (CFP)-tagged SGLT1 or CFP-tagged SGLT2 or no transfection in COS-7 cells, immunoblot of lysates using Novus anti-SGLT2 antibody, without and with pre-absorption of the antibody with the recombinant SGLT2 antigen peptide; immunoblot for CFP was used as a loading control and served to validate the molecular weight position of the CFP-SGLT2 specific signal. (**c**) Immunolabeling of mouse kidney section using Novus anti-SGLT2 antibody, without and with pre-absorption of the antibody with the recombinant SGLT2 antigen peptide, shown at low (*upper*) and high (*lower*) magnification; scale bars, 100 and 50 µm. (**d**) Immunolabeling of mouse kidney section using Novus anti-SGLT2 antibody, followed by processing for RNAScope for *Slc5a2*; individual labelings and the merged image are shown; scale bar, 50 µm. (**e**) Immunolabeling of mouse choroid plexus using Novus anti-SGLT2 antibody, without and with pre-absorption of the antibody with the recombinant SGLT2 antigen peptide; scale bar, 100 µm.
